# The Emerging Prevalence of Obesity within Families in Europe and its Associations with Family Socio-Demographic Characteristics and Lifestyle Factors; A Cross-Sectional Analysis of Baseline Data from the Feel4Diabetes Study

**DOI:** 10.3390/nu15051283

**Published:** 2023-03-04

**Authors:** George Siopis, George Moschonis, Kyriakos Reppas, Violeta Iotova, Yuliya Bazdarska, Nevena Chakurova, Imre Rurik, Anette Si Radó, Greet Cardon, Marieke De Craemer, Katja Wikström, Päivi Valve, Luis A. Moreno, Pilar De Miguel-Etayo, Konstantinos Makrilakis, Stavros Liatis, Yannis Manios, on behalf of the Feel4Diabetes-Study Group

**Affiliations:** 1 Department of Food, Nutrition and Dietetics, School of Allied Health, Human Services and Sport, La Trobe University, Melbourne, VIC 3086, Australia; 2 Institute for Physical Activity and Nutrition, Deakin University, Geelong, VIC 3220, Australia; 3 Department of Nutrition and Dietetics, School of Health Science and Education, Harokopio University, 17671 Athens, Greece; 4 Department of Endocrinology, Medical University Sofia, 1431 Sofia, Bulgaria; 5 Department of Family Medicine, Semmelweis University, 1085 Budapest, Hungary; 6 Hungarian Society of Nutrition, 1088 Budapest, Hungary; 7 Department of Nursing and Midwifes, Faculty of Health Sciences, University of Debrecen, 4400 Debrecen, Hungary; 8 Department of Movement and Sports Sciences, Faculty of Medicine and Health Sciences, Ghent University, 9000 Gent, Belgium; 9 Department of Rehabilitation Sciences, 9000 Ghent, Belgium; 10 Research Foundation Flanders, Ghent University, 9000 Ghent, Belgium; 11 The Department of Public Health and Welfare, Finnish Institute for Health and Welfare, 00271 Helsinki, Finland; 12 Growth, Exercise, Nutrition and Development (GENUD) Research Group, Instituto Agroalimentario de Aragón (IA2), Universidad de Zaragoza, Instituto de Investigación Sanitaria de Aragón (IIS Aragón), 50009 Zaragoza, Spain; 13 Centro de Investigación Biomédica en Red de Fisiopatología de la Obesidad y Nutrición (CIBEROBN), Instituto de Salud Carlos III, 28029 Madrid, Spain; 14 First Department of Propaedeutic Medicine, Laiko General Hospital, Athens Medical School, National and Kapodistrian University of Athens, 11527 Athens, Greece; 15 Institute of Agri-Food and Life Sciences, Hellenic Mediterranean University Research Centre, 71410 Heraklion, Greece

**Keywords:** BMI, community intervention, lifestyle intervention, overweight prevention, school, SES, socio-economic risk factors, T2D, T2DM, weight

## Abstract

The Feel4Diabetes study is a type 2 diabetes prevention program that recruited 12,193 children [age: 8.20 (±1.01) years] and their parents from six European countries. The current work used pre-intervention data collected from 9576 children–parents pairs, to develop a novel family obesity variable and to examine its associations with family sociodemographic and lifestyle characteristics. Family obesity, defined as the presence of obesity in at least two family members, had a prevalence of 6.6%. Countries under austerity measures (Greece and Spain) displayed higher prevalence (7.6%), compared to low-income (Bulgaria and Hungary: 7%) and high-income countries (Belgium and Finland: 4.5%). Family obesity odds were significantly lower when mothers (OR: 0.42 [95% CI: 0.32, 0.55]) or fathers (0.72 [95% CI: 0.57, 0.92]) had higher education, mothers were fully (0.67 [95% CI: 0.56, 0.81]) or partially employed (0.60 [95% CI: 0.45, 0.81]), families consumed breakfast more often (0.94 [95% CI: 0.91 0.96]), more portions of vegetables (0.90 [95% CI: 0.86, 0.95]), fruits (0.96 [95% CI: 0.92, 0.99]) and wholegrain cereals (0.72 [95% CI: 0.62, 0.83]), and for more physically active families (0.96 [95% CI: 0.93, 0.98]). Family obesity odds increased when mothers were older (1.50 [95% CI: 1.18, 1.91]), with the consumption of savoury snacks (1.11 [95% CI: 1.05, 1.17]), and increased screen time (1.05 [95% CI: 1.01, 1.09]). Clinicians should familiarise themselves with the risk factors for family obesity and choose interventions that target the whole family. Future research should explore the causal basis of the reported associations to facilitate devising tailored family-based interventions for obesity prevention.

## 1. Introduction

Overweight and obesity is the largest non-communicable disease pandemic affecting more than 1.9 billion adults and nearly four hundred million children and adolescents [1]. Overweight and obesity are risk factors for other non-communicable diseases, such as type 2 diabetes (T2D), cardiovascular disease, and certain forms of cancer, that collectively account for more than 40 million deaths per annum [2]. Moreover, overweight and obesity are also a risk factor for coronavirus disease-19 (COVID-19), with the Centers for Control and Disease Prevention (CDC) in the US reporting that 4 in 5 hospitalised people in the US with COVID-19 have overweight or obesity [3], and the World Obesity Federation emphasising a “dramatic correlation” between COVID-19 mortality and obesity [4].

The prevalence of obesity worldwide is on the rise, having nearly tripled in thirty years between 1975 and 2016 [1]. Childhood overweight and obesity rates are increasing at an alarming rate, with the prevalence of overweight and obesity among children and adolescents aged 5–19 years having risen from 4% in 1975 to more than 18% in 2016 [1]. Obesity exhibits a steeper trend than that of overweight in children and adolescents, with 8% of boys and 6% of girls having obesity in 2016, compared to less than 1% in 1975 [1].

An association between the obesity status of children and that of parents and certain behaviours of parents has been previously reported [5]. Preliminary evidence shows that children are more likely to have obesity if their parents have obesity or if their parents follow unhealthy lifestyle practices, such as an unhealthy diet and not being physically active enough [6,7]. Despite the important role of the social and physical environment within the family in shaping behaviours that affect energy balance and consequently the weight status of family members, research has primarily focused on the identifications of obesity risk factors only at an individual level. A previous study from Finland reported the association between parental body mass index (BMI), family structure, socioeconomic factors, and childhood overweight [8]. However, to the best of our knowledge, no previous study has assessed these associations in more than one country. Investigating these associations in diverse demographic and socioeconomic settings is important to understand the risk factors for childhood overweight and obesity and to allow for an informed approach to devising effective and feasible obesity prevention strategies.

Previous studies have explored obesity within families, and how overweight and obesity of the parents can affect the weight of the children, but either the sample sizes were small or they used single country data [9,10,11]. This study used the pre-intervention demographic and anthropometric data collected from children and their parents participating in the Feel4Diabetes study [12], to report the prevalence of family obesity and examine the potential associations of family obesity with the demographic characteristics and lifestyle factors of residents from the six European countries that participated in the Feel4Diabetes study.

## 2. Materials and Methods

Detailed information on the materials and methods used to recruit the study sample and collect data in the Feel4Diabetes study, as well as on the validity and/or reliability of the tools and/or procedures followed is presented elsewhere [13,14].

### 2.1. Study Design and Sampling Procedures

The Feel4Diabetes study (http://feel4diabetes-study.eu/, NCT02393872) was a large-scale community-based, family-involved study that aimed to promote a healthy lifestyle, including healthy eating and increased physical activity, in families from six European countries, namely Belgium, Bulgaria, Finland, Greece, Hungary, and Spain. The Feel4Diabetes intervention was implemented during two school years (2016–2018). The study was conducted within selected socioeconomic areas in the participating European countries and the recruitment was based on a standardised, multi-stage sampling procedure, [12]. Specifically, in low to middle income countries, i.e., Bulgaria and Hungary, all the municipalities within the participating regions were eligible for recruitment, while in high-income countries, i.e., Belgium, Finland, Greece, and Spain, families within low SES municipalities were recruited [15]. In high-income countries, low SES municipalities were defined as those with the lowest educational level and/or the highest unemployment rates, as retrieved from official resources and local authorities within each country. To be considered for inclusion, children and their parents had to be living in low socioeconomic areas or belong to vulnerable subgroups of the population, or both. Children also had to be attending one of the first three grades of compulsory education. There were no exclusion criteria other than those directly opposite to the inclusion criteria. The details of the study design and sampling procedures have already been published [12]. In brief, primary schools in the selected municipalities served as entry-points to communities. Parents of children in the first three grades of these schools were invited to participate in the study. Regarding sample size, a sample of 600 families per treatment arm was required to achieve a statistical power greater than 80% (at a two-sided 5% significance level) for reducing screen time by 0·2 h/d in children within 8 months. However, to account for an estimated dropout rate of about 20%, there was an aim to recruit a total number of about 9000 families in the six participating countries.

### 2.2. Ethics Approvals and Consent Forms

The Feel4Diabetes study adhered to the Declaration of Helsinki and the conventions of the Council of Europe on human rights and biomedicine [16]. Prior to initiating the study, researchers in participating countries obtained ethics approval from local authorities. Participants were presented with a detailed description of the study and asked to fill in and sign consent forms for their participation, and were given the chance to withdraw from the study at any point.

### 2.3. Data Collection

Data were collected at baseline (2016), and during the first (2017) and the second year (2018) of the program by rigorously trained researchers, who were trained as part of a central training which aimed to standardize researchers and minimize intra- and inter-observer variability. [14]. In brief, the central training of the Feel4Diabetes intervention ensured that the anthropometric and blood pressure data collected from study participants were valid and comparable [14]. Furthermore, the questions used to assess children’s and parents’ dietary and physical-activity-related behaviours had an acceptable test–retest reliability, with several questions showing excellent reliability (Intraclass Correlation Coefficients > 0.81) [13].

#### 2.3.1. Socio-Demographic Characteristics

Information on the socio-demographic characteristics (e.g., age, gender, race, education, marital and employment status) of parents and their children within the examined families was collected via self-reported questionnaires. Continuous variables were dichotomised as follows: age: <45 years vs. ≥45 years, education: <9 years, 9–14 years, and >14 years. Forty-five years is generally the age that middle age starts [17,18], although the exact age is disputed [19]. Nine years is the duration of compulsory education in most European education systems [20]. The categorical variable “occupation” was trichotomised as “employed full-time”, “employed part-time”, and “unemployed/other”.

#### 2.3.2. Anthropometry

Standing height was measured without shoes and was recorded to the nearest tenth of a centimetre (i.e., 0.1 cm) using telescopic stadiometers: SECA 213, SECA 214, SECA 217, and SECA 225. Body weight was measured with light clothing and without shoes and recorded to the nearest 0.1 kg. The equipment for measuring body weight included electronic weight scales: SECA 813 and SECA 877. Body mass index (BMI) was calculated according to the WHO formula and its reporting followed the WHO classification for adults [1] and for children [21,22]. Family obesity was defined as the presence of obesity in at least two out of the three family members participating in the Feel4Diabetes study (i.e., both parents or the child and any of the two parents).

#### 2.3.3. Lifestyle Factors

Parents filled in questionnaires about their and their children’s energy balance related behaviours, which were relevant to dietary intake, physical activity, and screen time. The reliability of the questionnaires regarding these lifestyle behaviours was evaluated in a pilot study and was found to be acceptable [13].

##### Dietary Intake

Consumption of water, fruit and vegetables, dairy (unsweetened or sweetened), cereals (low fibre or wholegrain), soft drinks (with or without sugar), sweets, savoury snacks, fast food, and breakfast were assessed with the use of a Food Frequency Questionnaire (FFQ). The general question in the FFQ was: “Indicate how often you (parent) and your child consume: water, soft drinks with or without added sugar, fruit/berries (fresh or frozen), fruit and berries (canned or dried), vegetables, dairy (sweetened or unsweetened), sweets, cereals (low fibre or wholegrain), salty snacks/fast-food, and breakfast”. Depending on the food items, answer options were less than 1 time or day/week, 1 or 2 times or days/week, 3 or 4 times or days/week, 5 or 6 times or days /week, as well as 1 or 2 portions or cups/day, 3 or 4 portions or cups/day, 5 or 6 portions or cups/day, >6 portions or cups/day. These categorical variables were then recoded into numerical ones and were used to calculate the sum of daily consumption of all aforementioned food items by all family members. Outliers for the total daily food consumption (defined as values above three standard deviations from the mean) were capped and reassigned the value of the mean plus three standard deviations. The daily breakfast consumption was measured by the following questions. “How many days do you/does your child usually eat breakfast?” separately for weekdays and weekend days. The number of days consuming breakfast on weekdays and weekend days were summed for all family members.

##### Physical Activity and Screen Time

Moderate-to-vigorous physical activity (MVPA) was subjectively measured by the following questions: “How many days during the last week did you (parent) spend in MVPA for a total of at least 30 min per day?” and “How many days during the last week did your child spend in MVPA for a total of at least 1 h per day?”. Parents’ and children’s screen-time behaviour during the week was assessed by the following question: “About how many hours per day do you (parent) and your child usually devote to screen-activities (excluding school/work)?”. The questions that were used to collect this data are two separate ones (one for the parent and one for the child). Answer options (categorical values) were expressed in hours per day. Answers options: None, <30 min/day, 30 min to <1 h/day, 1 to <2 h/day, 2 to <3 h/day, …, ≥7 h/day. Afterwards, these categorical values were recoded into continuous variables, which were expressed as number of days per week when each family member meets physical activity recommendations, or as hours per day of screen time. Each one of these continuous variables was summed for all family members.

### 2.4. Statistical Analysis

All statistical analyses were performed using the Statistical Package for Social Sciences (SPSS Inc., Chicago, IL, USA), version 25.0. The normality of the distribution of continuous variables was tested by the Kolmogorov–Smirnov test. Normally distributed continuous variables are presented as means and standard deviations (SD), while non-normally distributed ones are presented as medians and interquartile ranges (IQR). Categorical variables are presented as percentages (%).

Between-group differences of continuous variables were tested using either one-way analysis of variance (ANOVA) or the non-parametric Kruskal–Wallis test for normally and non-normally distributed variables, respectively. The significance of the association between categorical variables was examined using the chi-squared (χ²) test. Stratified analyses were carried out using an economic classification of countries at the time of submission of the study protocol (2014–2015), i.e., “low-income” (Bulgaria and Hungary), “under austerity measures” (Greece and Spain), and “high-income” (Belgium and Finland). Regarding the characterization of Greece and Spain as counties under austerity measures, this is a definition that was based on historical financial data indicating that both Greece and Spain faced a sovereign debt crisis following the world financial crisis of 2007–2008 [23]. This resulted in a series of reforms and austerity measures that led to recession, loss of income, and a negative impact on both countries’ healthcare systems in the following years, which coincided with the time period when the Feel4Diabetes study was conducted.

Univariate logistic regression analyses were initially carried out to examine the crude associations between family obesity (dependent variable) and family socio-demographic characteristics and lifestyle factors (independent variables) for the total sample and by economic classification of countries. Those variables that were found to be significantly associated with family obesity at a univariate level, were then all entered to relevant multivariate logistic regression models, which allowed the examination of potentially direct (i.e., independent from other potential confounders) significant associations with family obesity. The existence of multicollinearity was tested via the examination of the correlation matrix and the variance inflation factor (VIF) for all independent variables. The correlation coefficients and the VIF value did not relevel any multicollinearity that could bias the results of the multivariate regression models. The Bonferroni correction was applied to control for multiple comparisons. All reported *p*-values were two-tailed, and the level of statistical significance was set at *p* < 0.05.

## 3. Results

**Figure 1:**
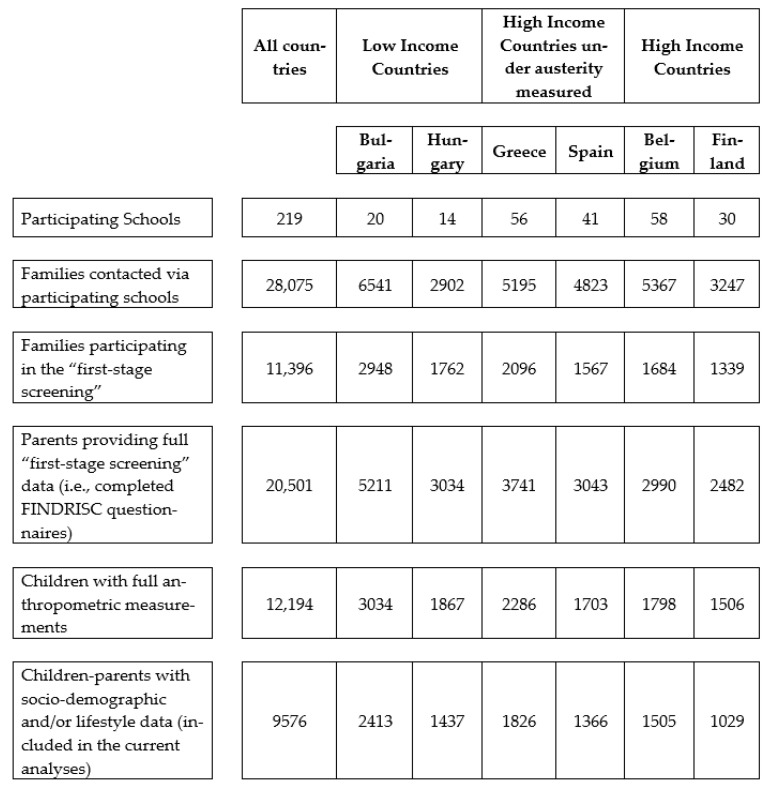
Flow chart of study participants in the Feel4Diabetes study.

The results presented in this section are derived from the statistical analyses conducted on children–parents pairs with full data on the examined variables (*n* = 9576). Figure 1 shows the participant flow.

### 3.1. Prevalence of Family Obesity in the Total Sample, by Country’s Economic Classification, and by Country

**Figure 2:**
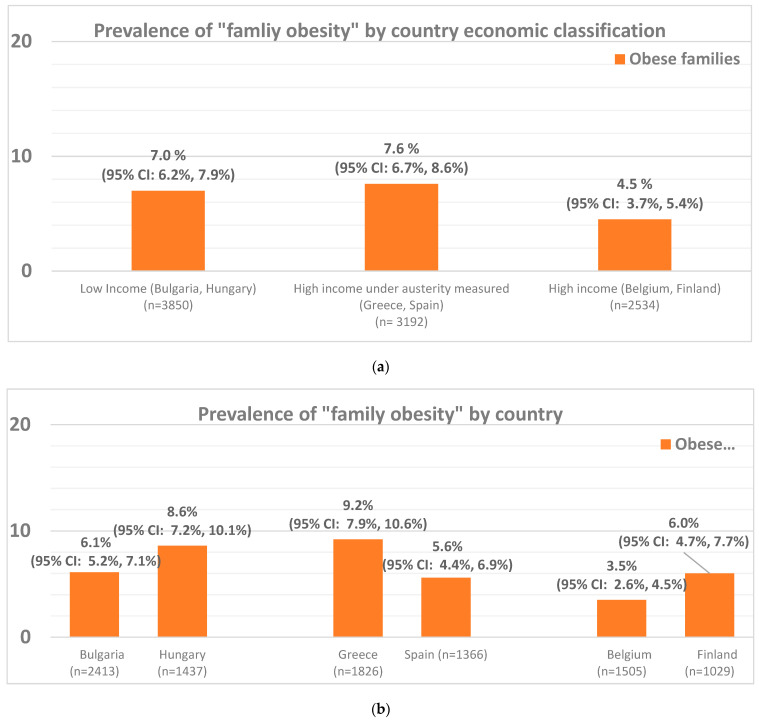
Prevalence of family obesity in the total sample, by country’s economic classification (**a**) and by country (**b**). *p*-value derived from χ^2^ test. Percentages that share the same superscript letter are statistically significantly different (*p* < 0.05) according to pairwise comparisons between countries and economic classification of countries. Countries classified in three economic categories at the time the data were collected, as “low-income” (Bulgaria and Hungary), “high-income, under austerity measures” (Greece and Spain), and “high-income” (Belgium and Finland). Family obesity was defined as the presence of obesity in at least two out of the three family members participating in the Feel4Diabetes study (i.e., both parents or the child and any of the two parents).

Figure 2 presents the data on the prevalence of family obesity in the total sample by country’s economic classification and by country. Overall, nearly seven percent (*n* = 628 out of 9576; 6.6%) of participating families in the total sample had obesity. Countries under austerity measures displayed the highest family obesity rate (*n* = 244 out of 3192; 7.6%) followed by low-income countries (*n* = 270 out of 3850; 7%), with high-income countries displaying the lowest prevalence of family obesity (*n* = 114 out of 2534; 4.5%) (*p* < 0.001 as per the data resented in Table 1). Further stratification by country revealed that the highest family obesity prevalence was seen in the south–east part of Europe, with Greece displaying the highest prevalence at 9.2% (*n* = 168 out of 1826), followed by Hungary at 8.6% (*n* = 123 out of 1437), and Bulgaria at 6.1% (*n* = 147 out of 2413). The north–west part of Europe displayed lowest family obesity rates, with Finland at 6% (*n* = 62 out of 1029), Spain at 5.6% (*n* = 76 out of 1366) and Belgium at 3.5% (*n* = 52 out of 1505). Table 1 also presents the prevalence of family obesity and all combinations of the presence of obesity in different family members that were used in the definition of family obesity (i.e., two obese parents/non-obese child; One obese parent/ obese child; Two obese parents/obese child) by country’s economic classification. According to this data the prevalence of obesity in families with one obese parent and an obese child was found to be significantly higher in high-income countries under austerity measures (*n* = 109 out of 3192; 3.4%) and low-income countries (*n* = 131 out of 3850; 3.4%) compared to high-income countries (*n* = 33 out of 2534; 1.3%) (*p* < 0.001 as per the data resented in Table 1). No other statistically significant differences in the prevalence of the different definition categories of family obesity were observed among countries with economic classification.

### 3.2. Socio-Demographic Characteristics of Families in the Total Sample and by Economic Classification of Countries

Table 1 presents the socio-demographic characteristics of participating families. Approximately, half of the children in the study were boys and half girls. The vast majority of parents were younger than 45 years old (90.4% of mothers and 77.7% of fathers). High-income countries displayed overall the highest education percentages of parents (63.3% of mothers and 48.3% of fathers having completed more than 14 years of education) along with the highest full-time employment rates for them, with the exception of mothers that displayed a marginally higher full-time employment rate in low-income countries compared to high-income ones (62% vs. 60.9%, respectively) and fathers displaying a slightly higher percentage for education in countries “under austerity measures” (50.8%). Conversely, low-income countries displayed the lowest education status for mothers and fathers (equally low with countries under austerity measures—both at 11%), along with the highest unemployment rates for fathers (19.2%) and second highest for mothers (32.1%, the highest was seen in countries under austerity measures at 35.5%).

### 3.3. Dietary Intake, Physical Activity Levels, and Screen Time of Families in the Total Sample and by Economic Classification of Countries

Table 2 presents the data on participants’ dietary intake, physical activity levels, and screen time. Participating families from countries under austerity measures displayed the least healthy behaviours (e.g., lower vegetable and fruit intake, higher sweetened dairy intake, higher low-fibre cereal and lower wholegrain cereal intake, less physical activity) compared to low-income and high-income countries (*p* < 0.001). However, the average screen time in high-income countries under austerity measures was lower compared to the rest of the countries falling under the other two economic classification categories (*p* < 0.001). On the contrary, participating families from high-income countries reported consuming more vegetables and wholegrain cereal, along with meeting the recommended physical activity levels more frequently, compared to participants from low-income countries (*p* < 0.001) and the ones in high-income countries under austerity measures (*p* < 0.001).

### 3.4. Associations between Sociodemographic Characteristics and Family Obesity

Table 3 presents the univariate logistic regression analyses examining the crude associations between each one of the family sociodemographic characteristics and family obesity for the total sample and by country’s economic classification. Compared to families with parents younger than 45 years of age, there was a 50% higher likelihood of family obesity when the mother’s age was equal to or older than 45 years in the total sample. The likelihood of family obesity was higher in low-income (55% higher) and high-income countries (173% higher), but not in high-income countries under austerity measures when the mother’s age was equal to or older than 45 years, and when the father’s age was equal to or older than 45 years only in high-income countries (123% higher). Regarding education, there was a lower likelihood of family obesity in families with parents that had completed more years of education, in those with parents having completed more than 14 years of education showing the lowest likelihood of family obesity (76% less for fathers and 66% less for mothers in high-income countries), followed by families with parents having completed 9–14 years (53% less for fathers only, in high-income countries) compared to having completed less than nine years of education. This trend was seen in all countries regardless of their economic classification, but its values were more significant for fathers than for mothers. On the other hand, more significant associations with family obesity were observed for the occupation of the mothers, with a significantly lower likelihood of family obesity seen when the mother was full-time (33% less likelihood) or part-time employed (40% less likelihood), compared to unemployed in the total sample, and a 64% lower likelihood of family obesity when the mother was full-time and 50% lower likelihood when she was part-time employed in high-income countries, and a 46% lower likelihood when the mother was part-time employed in high-income countries under austerity measures. Significantly lower odds of family obesity prevalence were also seen when the father was full-time employed in high-income countries (48% lower likelihood) and in high-income countries under austerity measures (38% lower likelihood).

### 3.5. Associations between Lifestyle Factors and Family Obesity

Table 4 presents the univariate logistic regression analyses examining the crude associations between lifestyle factors and family obesity for the total sample and by country’s economic classification. An increased likelihood of family obesity was observed in low-income countries with higher soft drink and savoury snack consumption, with the odds increasing by 10% for every portion of “diet” soft drink and by 8% for every portion of savoury snacks consumed per day, respectively. Furthermore, in high-income countries, the likelihood of family obesity increased by 20% for every portion of “diet” soft drink consumed per day and by 48% for every time per day that sweetened dairy was consumed. Reduced odds for family obesity were seen in high-income countries, by 8% for every day of the week that breakfast was consumed, and interestingly, by 18% for every portion of sweets per day consumed. In high-income countries under austerity measures, the chances of family obesity increased by 15% for every cup of water consumed per day and by 10% for every hour spent in front of a screen per day. On the other hand, the chances for family obesity decreased by 21% for every time per day sweetened dairy were consumed, by 8% for every day of the week that breakfast was consumed, and by 9% for every day of the week that the family was meeting physical activity recommendations.

### 3.6. Associations between Sociodemographic Characteristics and Lifestyle Factors with Family Obesity

Table 5 presents the multivariate logistic regression analyses examining the associations between those sociodemographic characteristics and lifestyle factors that were found to have statistically significant odds ratios at a univariate level, and family obesity for the total sample and by country’s economic classification. In the total sample as well as in low-income countries and high-income countries under austerity measures, the likelihood of family obesity was lower for fathers who had completed more years of education. Furthermore, in high-income countries, the status of the education of the father did not produce statistically significant results but the employment status of the mother did, with the odds for family obesity being reduced by 45% when the mother was full-time employed.

Regarding lifestyle factors, an increase of 11% in the chances of family obesity was seen in the total sample (24% in high-income countries under austerity measures and 6% in low-income countries) for every cup of water consumed per day. Higher odds for family obesity were also seen in the total sample, increasing by 14% for every portion of savoury snacks consumed per day, and in high-income countries, increasing by 29% for every portion of “diet” soft drinks consumed per day. On the other hand, a reduction in the likelihood of family obesity by 5% in the total sample (9% in high-income under austerity measures countries and 5% in low-income countries) was observed for every day of the week that the family was meeting the physical activity recommendations. Interestingly, a 13% reduction in the total sample (29% in low-income countries) for the chances of family obesity was observed for every portion of sweets consumed per day.

## 4. Discussion

This study utilised a rich dataset on the anthropometric characteristics of parents and children living in six European countries, in order to report the prevalence of family obesity and also to examine its potential associations with the participants’ self-reported socio-demographic and lifestyle characteristics. Overall, one in every fifteen participating families had obesity. The prevalence of family obesity, defined as the presence of obesity in at least two out of the three family members participating in the Feel4Diabetes study (i.e., both parents or the child and any of the two parents), was higher in high-income countries under austerity measures, with one in thirteen families, and lower in high-income countries, with one in twenty-two families displaying obesity. Our findings are in agreement with previous research that has shown a disproportional distribution of the prevalence of non-communicable disease, with lower socioeconomic areas displaying higher prevalence rates [24,25,26]. Lower socioeconomic status is often associated with a lower overall education status, lower health literacy, and lower employment rates [27]. Indeed, our study demonstrated strong associations between the education status of the parents and family obesity, with the latter exhibiting its highest values when either parent had attained fewer years of education. Patients have previously reported that health literacy facilitates the necessary dietary changes towards achieving their health objectives [28]. In terms of employment, family obesity was lower when either parent was employed compared to being unemployed. Our finding agrees with previous research that has highlighted lower rates of engagement with health services for unemployed people with type 2 diabetes [29,30].

When examining each country on its own, the highest family obesity prevalence was observed in Greece, where one in eleven families displayed obesity. This finding is in agreement with previous research which has shown that the social and physical environment that shapes energy-balance-related behaviours in the family is less supportive in Greece and low-income European countries, compared to high-income European countries [31]. Children’s social environment, which primarily consists of family (i.e., parents and siblings), peers, and teachers, determines their health behaviour, mainly via modelling, encouragement, support, rule setting, and rewarding. In this social context, children can adopt eating and sedentary behaviour from their parents and peers, can be encouraged or rewarded for eating fruits and vegetables by their parents or teachers, but also teased about these food choices by their peers. Physical environment, such as home, school, and neighbourhood, is of equal importance to the social one since it can also play a pivotal role in the adoption of certain health behaviours by children. In this context, it is known that availability and accessibility to certain foods, sports equipment and sports facilities are important factors in determining children’s obesity-related behaviour [32].

Family obesity prevalence was lower when the parents’ age was less than 45 years, compared to when it was equal to or older than 45 years. Younger parents tend to be more physically active and previous research has demonstrated lower childhood obesity rates in families with younger parents [33,34]. Parents are role models for young children and therefore younger parents that are more active may be positively influencing the health behaviour within the family, including more favourable physical activity and eating habits [6,7,35,36,37]. Our study showed increased family obesity odds with increased screen time. This can partially be explained via the association between increased screen time with reduced time spent being physically active [38,39], although this association has not always been found [40]. Another explanation relies on the finding that the longer the screen time, the higher the odds for binge-eating and overconsumption of food, even in the absence of hunger cues [41,42,43,44,45].

In terms of its associations with dietary intake, the odds for family obesity were reduced when breakfast was consumed more frequently. This is agreement with studies that have shown that skipping breakfast is correlated with obesity [46,47,48]. It remains to be determined if this association has a causative component, e.g., an amplified morning thermogenesis [49], or if it is due to, e.g., children having breakfast also being likely to adopt other healthy behaviours such as a healthy diet in general and physical activity. Interestingly, reduced odds for family obesity were observed in participants from high-income countries with an increased daily consumption of sweets. A possible explanation for this paradox may be that the overall energy balance in this population was favourable for a normal body mass index. Indeed, participants from high-income countries displayed the highest levels of physical activity, meaning that their increased energy expenditure may counteract the extra calories derived from sweets. A potential underreporting of sweets consumption by parents for their children (because, e.g., this is a “socially acceptable” answer) in low-income and in high-income countries under austerity measures cannot be excluded. Finally, different perceptions of what counts as sweets in the different countries (e.g., in some European countries, the consumption of cookies with milk might be considered as a nutritious snack, instead of eating a sweet with milk) could provide another explanation.

Regarding the associations between family obesity and other energy-balance-related behaviours, increased odds for family obesity were observed with an increased consumption of processed foods such as soft drinks, savoury snacks, and sweetened dairy. These findings are in agreement with the literature [50,51,52,53]. Several characteristics of processed foods have been described as obesogenic, including their increased energy content, their nutrient profile, their effect on distorting digestive hormone balance and inducing high-glycaemic responses, and their inclusion of cosmetic additives with pro-inflammatory and obesogenic properties such as carboxymethylcellulose and polysorbate-80 [51,53,54]. Such additives promote adipogenesis via interfering with the expression of genes that affect fatty acid oxidation and fat deposition within the adipocyte [55]. Moreover, their xenobiotic nature triggers reactive oxygen species (ROS) generation and the formation of lipid hydroperoxides that promote the accumulation of lipids in the tissues, leading to obesity [54,56,57].

The strengths of this study lie in the sample size and the robust data collection methods of the Feel4Diabetes study, including the standardised way that anthropometry measurements were conducted in the different study centres, which involved centrally trained research team members in order to minimise any inter-observed variability, and the validated questionnaires used to collect dietary, physical activity, and screen-time information [12,13]. Additionally, we performed a series of tests for a potential confounding effect, by including numerous socio-demographic and lifestyle variables in the regression models; however, as we have not exhausted all such variables, the possibility of confounding cannot be excluded. A limitation of the study design is that causal associations cannot be explored. Furthermore, missing data were not accounted for in the analyses. Moreover, the self-reporting of part of the collected data is prone to recall bias and social desirability. Finally, as Feel4Diabetes used school as an entry point to recruit participants, the results might not be applicable to single adults or families with no children at all or no primary school children. However, within the context of this study’s target population, the results can be generalised to the whole population of adults/families with children attending primary school, considering that the participation rate of the families was quite high [12].

## 5. Conclusions and Implications for Practice and Future Research

In conclusion, family obesity prevalence was higher in high-income countries under austerity measures and low-income countries compared to high-income countries. Reduced odds for family obesity were observed when the parents were younger, had completed more years of education, were employed, when breakfast was consumed more frequently, and when physical activity recommendations were met more frequently. The odds increased with the consumption of processed foods and with increased screen time. Clinicians should familiarise themselves with the risk factors for family obesity and choose obesity interventions that target the whole family rather than individuals. Future research should explore the causal basis of the reported associations to facilitate an insight into the most important risk factors for family obesity within different regions in Europe that will appropriately inform European and country-specific public health policy. Future research should also highlight the socio-demographic factors of families that are most in need of an intervention, as well as the energy-balance-related behaviours that need to become the target of appropriate family-based intervention programs in Europe. This knowledge will set the basis for developing more tailored and effective family-based interventions for the prevention of obesity within families.

## Figures and Tables

**Table 1:** Socio-demographic characteristics of families in the total sample and by economic classification of countries.

		Total Sample (*n* = 9576)	Economic Classification of Countries			
			Low-Income Countries (*n* = 3850)	High-Income Countries, under Austerity Measures (*n* = 3192)	High-Income Countries (*n* = 2534)	*p*-Value
Obesity (%)	Family Obesity *	6.6	7.0 ^c^	7.6 ^b^	4.5 ^b,c^	<0.001
	Two obese parents/non-obese child	2.7	2.7	2.9	2.5	0.703
	One obese parent/obese child	2.8	3.4 ^c^	3.4 ^b^	1.3 ^b,c^	<0.001
	Two obese parents/obese child	1.1	0.9	1.3	0.7	0.069
Child sex (%)	Boys	49.4	48.5	50.0	50.0	0.260
	Girls	50.6	51.5	50.0	50.0	
Age of Mother (%)	<45 years old	90.4	93.1 ^a^	85.6 ^a,b^	91.9 ^b^	<0.001
	≥45 years old	9.6	6.9 ^a^	14.4 ^a,b^	8.1 ^b^	
Age of Father (%)	<45 years old	77.7	81.3 ^a^	68.6 ^a,b^	83.3 ^b^	<0.001
	≥45 years old	22.3	18.7 ^a^	31.4 ^a,b^	16.7 ^b^	
Education of Mother (%) ^†^	<9 years	8.4	11.9 ^a,c^	7.7 ^a,b^	4.1 ^b,c^	<0.001
	9–14 years	35.3	34.3 ^a^	39.2 ^a,b^	32.6 ^b^	
	>14 years	51.9	53.8 ^c^	53.1 ^b^	63.3 ^b,c^	
Education of Father (%) ^†^	<9 years	9.7	10.9 ^c^	11.3 ^b^	6.2 ^b,c^	<0.001
	9–14 years	44.4	48.3 ^a^	37.9 ^a,b^	45.5 ^b^	
	>14 years	46.0	40.8 ^a,c^	50.8 ^a^	48.3 ^c^	
Occupation of Mother (%)	Unemployed/other #	29.5	32.1 ^a,c^	35.5 ^a,b^	19.3 ^b,c^	<0.001
	Employed full-time	57.5	62.0 ^a^	48.2 ^a,b^	60.9 ^b^	
	Employed part-time	13.1	6.0 ^a,c^	16.5 ^a,b^	19.8 ^b,c^	
Occupation of Father (%)	Unemployed/other #	14.1	19.2 ^a,c^	11.9 ^a,b^	9.4 ^b,c^	<0.001
	Employed full-time	81.5	75.4 ^a,c^	83.1 ^a,b^	88.6 ^b,c^	
	Employed part-time	4.3	5.4 ^c^	5.0 ^b^	2.0 ^b,c^	

*p*-value derived from χ^2^ test. Percentages that share the same superscript letter are statistically significantly different (*p* < 0.05), according to the pairwise comparisons between countries of different economic classifications. Countries classified in three economic categories at the time the data were collected, as “low-income” (Bulgaria and Hungary), “under austerity measures” (Greece and Spain), and “high-income” (Belgium and Finland). ^†^ Having completed less than 9, 9 to 14, or more than 9 years of education. # Never employed, or previously employed, or retired, etc. * Family obesity was defined as the presence of obesity in at least two out of the three family members participating in the Feel4Diabetes study (i.e., both parents or the child and any of the two parents).

**Table 2:** Dietary intake, physical activity levels, and screen time of families in the total sample and by economic classification of countries.

Lifestyle Factors		Economic Classification of Countries			
	Total Sample (*n* = 9576)	Low-Income Countries (*n* = 3192)	High-Income Countries, under Austerity Measures (*n*= 3850)	High-Income Countries (*n* = 2534)	*p*-Value *
	Median (IQR)	Median (IQR)	Median (IQR)	Median (IQR)	
Water (number of cups per day)	9.0 (7.0, 11.0)	9.0 (7.0, 12.0)	10.0 (7.0, 12.0)	7.0 (5.0, 9.0)	<0.001
Vegetables (number of portions per day)	2.3 (1.3, 3.0)	2.3 (1.3, 3.0)	1.6 (1.0, 2.3)	3.0 (1.7, 3.0)	<0.001
Fruits (number of portions per day)	2.9 (1.7, 3.7)	3.0 (1.7, 4.0)	2.4 (1.6, 3.4)	3.0 (1.7, 3.4)	<0.001
Dairy–unsweetened (number of times per day)	1.0 (0.5, 1.6)	1.0 (0.4, 1.3)	1.2 (0.7, 1.8)	1.2 (0.7, 1.8)	<0.001
Dairy–sweetened (number of times per day)	0.4 (0.0, 1.0)	0.4 (0.0, 1.0)	0.5 (0.0, 1.0)	0.4 (0.0, 0.8)	<0.001
Cereals–low fibre (number of times per day)	0.5 (0.2, 1.0)	0.5 (0.2, 1.0)	0.7 (0.2, 1.2)	0.5 (0.2, 1.0)	<0.001
Cereals–wholegrain (number of times per day)	0.7 (0.0, 1.2)	0.4 (0.0, 1.0)	0.4 (0.0, 1.0)	1.2 (0.7, 1.6)	<0.001
Soft drinks–with sugar (number of portions per day)	0.2 (0.1, 0.7)	0.3 (0.1, 1.0)	0.3 (0.1, 0.6)	0.3 (0.1, 0.7)	<0.001
Soft drinks–diet (number of portions per day)	0.1 (0.1, 0.3)	0.1 (0.1, 0.1)	0.1 (0.1, 0.3)	0.1 (0.1, 0.4)	<0.001
Sweets (number of portions per day)	1.0 (0.6, 2.0)	1.3 (0.7, 2.3)	1.0 (0.6, 1.6)	1.0 (0.4, 2.0)	<0.001
Savoury snacks and fast food (number of portions per day)	0.3 (0.1, 0.7)	0.4 (0.1, 1.0)	0.3 (0.1, 0.4)	0.3 (0.1, 0.4)	<0.001
Breakfast (number of days per week)	14.0 (11.0, 14.0)	13.0 (13.0, 14.0)	14.0 (11.0, 14.0)	14.0 (14.0, 14.0)	<0.001
Meeting PA recommendations (number of days per week)	10.0 (7.0, 12.0)	10.0 (10.0, 13.0)	9.0 (6.0, 12.0)	11.0 (8.0, 13.0)	<0.001
Average screen time (number of hours per day)	3.3 (2.2, 4.9)	3.6 (2.4, 5.3)	3.0 (1.9, 4.3)	3.6 (2.5, 4.8)	<0.001

IQR: Interquartile range.* The *p*-value is derived from the Kruskal–Wallis test and highlights the statistically significant differences in the dietary intake, physical activity levels, and screen time in families from countries with different economic classifications. IQR = interquartile range (25–75%). Countries classified in three economic categories at the time the data were collected, as “low-income” (Bulgaria and Hungary), “under austerity measures” (Greece and Spain), “high-income” (Belgium and Finland). Considering that the outcome examined in the present study is family obesity, the relevant behavioural exposures (e.g., breakfast, meeting PA recommendations, and average screen time) were also examined at a family level.

**Table 3:** Univariate logistic regression analyses examining the associations between sociodemographic characteristics and family obesity for the total sample and by country’s economic classification.

Independent Variables		Dependent Variable: Family Obesity			
		Total Sample (*n* = 9576)	Economic Classification of Countries *		
			Low-Income Countries (*n* = 3192)	High-Income Countries, under Austerity Measures (*n* = 3850)	High-Income Countries (*n* = 2534)
		**OR (95% C.I.)**	**OR (95% C.I.)**	**OR (95% C.I.)**	**OR (95% C.I.)**
Age of Mother	<45 years old	1.00	1.00	1.00	1.00
	≥45 years old	**1.50 (1.18, 1.91)**	**1.55 (1.01, 2.40)**	1.09 (0.75, 1.57)	**2.73 (1.63, 4.58)**
Age of Father	<45 years old	1.00	1.00	1.00	1.00
	≥45 years old	1.18 (0.97, 1.42)	1.02 (0.74, 1.40)	0.91 (0.68, 1.21)	**2.23 (1.51, 3.47)**
Education of Mother ^†^	<9 years	1.00	1.00	1.00	1.00
	9–14 years	0.91 (0.69, 1.20)	1.16 (0.79, 1.70)	0.75 (0.48, 1.18)	0.72 (0.33, 1.57)
	>14 years	**0.42 (0.32, 0.55)**	**0.53 (0.36, 0.79)**	**0.36 (0.23, 0.57)**	**0.34 (0.16, 0.74)**
Education of Father ^†^	<9 years	1.00	1.00	1.00	1.00
	9–14 years	**0.72 (0.57, 0.92)**	1.01 (0.70, 1.48)	**0.66 (0.45, 0.96)**	**0.47 (0.26, 0.84)**
	>14 years	**0.31 (0.24, 0.41)**	**0.44 (0.29, 0.67)**	**0.27 (0.18, 0.40)**	**0.24 (0.13, 0.44)**
Occupation of Mother	unemployed/other #	1.00	1.00	1.00	1.00
	employed full-time	**0.67 (0.56, 0.81)**	0.87 (0.66, 1.13)	0.75 (0.55, 1.01)	**0.36 (0.23, 0.56)**
	employed part-time	**0.60 (0.45, 0.81)**	0.94 (0.54, 1.63)	**0.54 (0.34, 0.86)**	**0.50 (0.29, 0.85)**
Occupation of Father	unemployed/other #	1.00	1.00	1.00	1.00
	employed full-time	0.81 (0.64, 1.02)	1.20 (0.85, 1.69)	**0.62 (0.42, 0.92)**	**0.52 (0.30, 0.88)**
	employed part-time	1.38 (0.93, 2.04)	1.79 (1.03, 3.09)	0.90 (0.46, 1.77)	1.39 (0.49, 3.97)

OR: Odds Ratio; 95% C.I.: 95% Confidence Interval. Numbers in bold indicate statistically significant odds ratios. * Countries classified in three economic categories at the time the data were collected, as “low-income” (Bulgaria and Hungary), “under austerity measures” (Greece and Spain), “high-income” (Belgium and Finland). ^†^ Having completed less than 9, 9 to 14, or more than 9 years of education. # Never employed, or previously employed, or retired, etc.

**Table 4:** Univariate logistic regression analyses examining the associations between lifestyle factors and family obesity for the total sample and by country’s economic classification.

Independent Variables		Dependent Variable: Family Obesity		
	Total Sample (*n* = 9576)	Economic Classification of Countries *		
		Low-Income Countries (*n* = 3192)	High-Income Countries, under Austerity Measures (*n*= 3850)	High-Income Countries (*n* = 2534)
	**OR (95% C.I)**	**OR (95% C.I)**	**OR (95% C.I)**	**OR (95% C.I)**
Water (number of cups per day)	**1.09 (1.06, 1.12)**	1.04 (0.99, 1.08)	**1.15 (1.09, 1.22)**	1.05 (0.98, 1.12)
Vegetables (number of portions per day)	**0.90 (0.86, 0.95)**	0.94 (0.88, 1.01)	0.91 (0.83, 1.00)	0.89 (0.79, 1.02)
Fruits (number of portions per day)	**0.96 (0.92, 0.99)**	0.96 (0.91, 1.01)	0.93 (0.86, 1.00)	0.99 (0.90, 1.11)
Dairy–unsweetened (number of times per day)	1.03 (0.91, 1.18)	1.13 (0.91, 1.41)	0.98 (0.80, 1.21)	1.12 (0.82, 1.54)
Dairy–sweetened (number of times per day)	1.02 (0.89, 1.18)	1.12 (0.90, 1.39)	**0.79 (0.63, 0.98)**	**1.48 (1.06, 2.08)**
Cereals–low fibre (number of times per day)	1.11 (0.96, 1.28)	1.06 (0.85, 1.31)	0.98 (0.77, 1.23)	1.31 (0.92, 1.87)
Cereals–wholegrain (number of times per day)	**0.72 (0.62, 0.83)**	0.87 (0.68, 1.11)	0.80 (0.62, 1.03)	**0.72 (0.53, 0.99)**
Soft drinks–with sugar (number of portions per day)	1.05 (0.99, 1.09)	1.05 (1.00, 1.11)	0.95 (0.81, 1.13)	1.05 (0.90, 1.22)
Soft drinks–diet (number of portions per day)	**1.10 (1.03, 1.17)**	**1.10 (1.02, 1.19)**	1.02 (0.82, 1.28)	**1.20 (1.06, 1.36)**
Sweets (number of portions per day)	0.97 (0.91, 1.02)	0.99 (0.92, 1.06)	1.02 (0.90, 1.15)	**0.82 (0.68, 0.98)**
Savoury snacks and fast food (number of portions per day)	**1.11 (1.05, 1.17)**	**1.08 (1.02, 1.16)**	1.15 (0.98, 1.34)	1.21 (0.96, 1.53)
Breakfast (number of days per week)	**0.94 (0.91, 0.96)**	0.98 (0.94, 1.02)	**0.92 (0.89, 0.96)**	**0.92 (0.85, 0.99)**
Meeting PA recommendations (number of days per week)	**0.96 (0.93, 0.98)**	0.99 (0.95, 1.03)	**0.91 (0.87, 0.95)**	0.99 (0.93, 1.05)
Average screen time (number of hours per day)	**1.05 (1.01, 1.09)**	1.02 (0.96, 1.08)	**1.10 (1.04, 1.17)**	1.05 (0.95, 1.16)

OR: Odds Ratio; C.I: Confidence Interval. Numbers in bold indicate statistically significant odds ratios. * Countries classified in three economic categories at the time the data were collected, as “low-income” (Bulgaria and Hungary), “high-income, under austerity measures” (Greece and Spain), “high-income” (Belgium and Finland).

**Table 5:** Multivariate logistic regression analyses examining the associations between sociodemographic characteristics, lifestyle factors, and family obesity for the total sample and by country’s economic classification.

Independent Variables		Dependent Variable: Family Obesity			
		Total Sample (*n* = 9576)	Economic Classification of Countries *		
			Low-Income Countries (*n* = 3192)	High-Income Countries, under Austerity Measures (*n*= 3850)	High-Income Countries (*n* = 2534)
***Socio-demographics***		**OR (95% C.I)**	**OR (95% C.I)**	**OR (95% C.I)**	**OR (95% C.I)**
Age of Mother	<45 years old	1.00	1.00	1.00	1.00
	≥45 years old	1.50 (0.99, 2.28)	1.11 (0.55,2.27)	1.34 (0.63, 2.88)	2.1 (0.99, 4.65)
Age of Father	<45 years old	1.00	1.00	1.00	1.00
	≥45 years old	1.02 (0.75, 1.38)	1.08 (0.69, 1.69)	0.79 (0.45, 1.39)	1.46 (0.78, 2.74)
Education of Mother ^†^	<9 years	1.00	1.00	1.00	1.00
	9–14 years	1.08 (0.67, 1.75)	1.83 (0.80, 4.22)	0.66 (0.31, 1.39)	1.49 (0.44, 5.02)
	>14 years	0.79 (0.46, 1.33)	1.20 (0.48, 2.96)	0.79 (0.34, 1.88)	0.80 (0.22, 2.85)
Education of Father ^†^	<9 years	1.00	1.00	1.00	1.00
	9–14 years	0.71 (0.48, 1.06)	0.53 (0.26, 1.11)	0.73 (0.40, 1.35)	0.65 (0.29, 1.47)
	>14 years	**0.37 (0.24, 0.59)**	**0.24 (0.10, 0.54)**	**0.39 (0.17, 0.86)**	0.50 (0.20, 1.23)
Occupation of Mother	unemployed/other #	1.00	1.00	1.00	1.00
	employed full-time	0.86 (0.66, 1.12)	0.91 (0.62, 1.34)	0.89 (0.54, 1.45)	**0.55 (0.31, 0.98)**
	employed part-time	0.71 (0.47, 1.08)	0.65 (0.28, 1.49)	0.78 (0.34, 1.80)	0.67 (0.34, 1.32)
Occupation of Father	unemployed/other #	1.00	1.00	1.00	1.00
	employed full-time	0.89 (0.65, 1.22)	0.96 (0.62, 1.49)	0.66 (0.35, 1.26)	1.04 (0.50, 2.16)
	employed part-time	1.41 (0.81, 2.47)	0.90 (0.36, 2.20)	1.18 (0.45, 3.05)	3.21 (0.89, 11.5)
***Lifestyle factors***					
Water (number of cups per day)		**1.11 (1.06, 1.15)**	**1.06 (1.01, 1.12)**	**1.24 (1.12, 1.37)**	**1.24 (1.12, 1.37)**
Vegetables (number of portions per day)		0.95 (0.89, 1.02)	0.97 (0.87, 1.07)	0.96 (0.82, 1.11)	0.96 (0.82, 1.11)
Fruits (number of portions per day)		0.97 (0.92, 1.03)	0.97 (0.91, 1.05)	0.95 (0.83, 1.08)	0.95 (0.83, 1.08)
Dairy–sweetened (number of times per day)		0.90 (0.72, 1.13)	0.77 (0.54, 1.09)	0.88 (0.57, 1.35)	0.88 (0.57, 1.35)
Cereals–wholegrain (number of times per day)		0.91 (0.75, 1.11)	0.97 (0.70, 1.34)	0.92 (0.62, 1.37)	0.92 (0.62, 1.37)
Soft drinks–diet (number of portions per day)		1.08 (0.99, 1.18)	1.05 (0.94, 1.18)	0.77 (0.33, 1.83)	0.77 (0.33, 1.83)
Sweets (number of portions per day)		**0.87 (0.78, 0.97)**	0.92 (0.81, 1.05)	0.92 (0.71, 1.19)	0.92 (0.71, 1.19)
Savoury snacks and fast food (number of portions per day)		**1.14 (1.01, 1.28)**	1.12 (0.97, 1.29)	0.97 (0.64, 1.50)	0.97 (0.64, 1.50)
Breakfast (number of days per week)		1.01 (0.97, 1.04)	1.02 (0.97, 1.08)	0.99 (0.93, 1.06)	0.99 (0.93, 1.06)
Meeting PA recommendations (number of days per week)		**0.95 (0.92, 0.98)**	**0.95 (0.90, 0.99)**	**0.91 (0.86, 0.97)**	**0.91 (0.86, 0.97)**
Average screen time (number of hours per day)		1.03 (0.97, 1.08)	1.02 (0.95, 1.10)	1.03 (0.93, 1.14)	1.03 (0.93, 1.14)

OR: Odds Ratio; C.I: Confidence Interval. Numbers in bold indicate statistically significant odds ratios.* Countries classified in three economic categories at the time the data were collected, as “low-income” (Bulgaria and Hungary), “high-income, under austerity measures” (Greece and Spain), “high-income” (Belgium and Finland). ^†^ Having completed less than 9, 9 to 14, or more than 9 years of education. # Never employed, or previously employed, or retired, etc.

## Data Availability

The Feel4Diabetes study data are subjected to data sharing restrictions and are only available upon request from the coordinating team.
